# Healthcare Associated Infections of Methicillin-Resistant *Staphylococcus aureus*: A Case-Control-Control Study

**DOI:** 10.1371/journal.pone.0140604

**Published:** 2015-10-15

**Authors:** Zhenjiang Yao, Yang Peng, Xiaofeng Chen, Jiaqi Bi, Ying Li, Xiaohua Ye, Jing Shi

**Affiliations:** 1 Department of Epidemiology and Health Statistics, Guangdong Pharmaceutical University, Guangzhou, China; 2 Division of Infectious Diseases, The People’s Hospital of Meizhou, Meizhou, China; 3 Department of Environmental and School Health, Shajing Health Inspection Institute, Shenzhen, China; 4 Division of Environmental Health, Public Health Laboratory Center, Guangdong Pharmaceutical University, Guangzhou, China; 5 Department of Preventive Medicine, Hebei University of Chinese Medicine, Shijiazhuang, China; Curtin University, AUSTRALIA

## Abstract

**Background:**

Methicillin-resistant *Staphylococcus aureus* (MRSA) is one of the most widespread and dangerous pathogens in healthcare settings. We carried out this case-control-control study at a tertiary care hospital in Guangzhou, China, to examine the antimicrobial susceptibility patterns, risk factors and clinical outcomes of MRSA infections.

**Methods:**

A total of 57 MRSA patients, 116 methicillin-susceptible *Staphylococcus aureus* (MSSA) patients and 102 *S*. *aureus* negative patients were included in this study. We applied the disk diffusion method to compare the antimicrobial susceptibilities of 18 antibiotics between MRSA and MSSA isolates. Risk factors of MRSA infections were evaluated using univariate and multivariate logistic regression models. We used Cox proportional hazards models and logistic regression analysis to assess the hospital stay duration and fatality for patients with MRSA infections.

**Results:**

The MRSA group had significantly higher resistance rates for most drugs tested compared with the MSSA group. Using MSSA patients as controls, the following independent risk factors of MRSA infections were identified: 3 or more prior hospitalizations (OR 2.8, 95% CI 1.3–5.8, *P* = 0.007), chronic obstructive pulmonary disease (OR 5.9, 95% CI 1.7–20.7, *P* = 0.006), and use of a respirator (OR 3.6, 95% CI 1.0–12.9, *P* = 0.046). With the *S*. *aureus* negative patients as controls, use of a respirator (OR 3.8, 95% CI 1.0–13.9, *P* = 0.047) and tracheal intubation (OR 8.2, 95% CI 1.5–45.1, *P* = 0.016) were significant risk factors for MRSA infections. MRSA patients had a longer hospital stay duration and higher fatality in comparison with those in the two control groups.

**Conclusions:**

MRSA infections substantially increase hospital stay duration and fatality. Thus, MRSA infections are serious issues in this healthcare setting and should receive more attention from clinicians.

## Introduction

Methicillin-resistant *Staphylococcus aureus* (MRSA) has spread throughout the world and has become one of the most frequent bacteria among healthcare-associated infections since it was first identified in 1961 [1–3]. MRSA can cause a number of life-threatening infections, such as septic shock, endocarditis, and severe pneumonia [4]. MRSA infections are closely linked to increased mortality, extra length of hospital stay and excess costs [5–8].

The spread of MRSA is also a serious issue in China. The proportion of MRSA among all *S*. *aureus* isolates has reached 50–70% based on former studies [9–11]. Due to the terrible prognosis of MRSA infections [7, 8], epidemiological information is urgently needed to help prevent and control these infections. However, recent systematic epidemiological surveys of hospital-acquired MRSA are lacking in Guangzhou, one of the largest cities in Southern China. Thus, we launched the current study to elucidate the antimicrobial susceptibility patterns, risk factors and clinical outcomes of MRSA infections in a tertiary care hospital in Guangzhou, China.

## Materials and Methods

### Setting and study design

The present study was carried out in a 1000-bed tertiary care hospital in Guangzhou, and patients admitted from January 2013 to December 2013 were included.

The enrolled *S*. *aureus* culture-positive patients were those for whom an *S*. *aureus* strain was first obtained from clinical samples at least 48 hours after admission. *S*. *aureus* patients were categorized into the MRSA group if their *S*. *aureus* isolates were positive for the *mec*A gene and resistant to oxacillin; otherwise, they were classified as methicillin-susceptible *Staphylococcus aureus* (MSSA) patients. Patients who were negative for *S*. *aureus* infections throughout hospitalization formed the *S*. *aureus* negative group. Patients were excluded from our study if they were discharged from the hospital within 48 hours.

Our study was designed to include three separate investigations. One compared the antimicrobial susceptibility profiles between the MRSA group and MSSA group. We also illustrated the risk factors of MRSA infections using two separate case-control studies: MRSA versus MSSA and MRSA versus *S*. *aureus* negative. Finally, we explored the impact of MRSA infections on the patients’ hospital stay duration and fatality.

Data were extracted from patients’ electronic medical records and included age, gender, ward, type of specimen, history of hospitalization, surgery, intensive care unit (ICU) admission, underlying illness, use of immunosuppressive drugs and antibiotics as well as treatment with invasive procedures during hospitalization, death versus survival, and length of hospital stay

Our research was conducted according to the Declaration of Helsinki. Our research was retrospective and only involved data obtained from electronic medical records and isolated strains. The doctors in the hospital anonymized the patients' identifying information, and the information was inaccessible to the authors throughout the data collection and data analysis process. Formal ethics approval from the Ethics Committee at Guangdong Pharmaceutical University and written/oral consent from the patients were not obtained.

### Laboratory methods

*S*. *aureus* isolates were identified using the Vitek 32 microbial identification system (bioMerieux, France). Antimicrobial susceptibility testing was conducted using the Kirby-Bauer disc diffusion and E-test methods following the guidelines of the Clinical and Laboratory Standard Institute [12]. In total, *S*. *aureus* strains were tested for susceptibility to 18 antibiotics, including gentamicin, imipenem, ciprofloxacin, levofloxacin, penicillin, vancomycin, linezolid, amoxicillin, ampicillin, oxacillin, sulfamethoxazole/trimethoprim, clindamycin, quinupristin/dalfopristin, rifampicin, chloramphenicol, tetracycline, cefazolin, and erythromycin. The E-test method was applied for vancomycin and oxacillin, and the disc diffusion method was used for the other agents.

### Statistical analysis

The sensitivities of the isolates to single drug agents were compared between the MRSA and MSSA groups using the chi-square test. Univariate and multivariate logistic regression models were used to explore risk factors for MRSA healthcare-associated infections. Variables with *P* values < 0.1 in the univariate logistic regression were introduced into a stepwise forward multivariate logistic regression model. Odds ratios (ORs) along with 95% confidential intervals (CIs) were calculated to assess the strengths of all associations. The Wald test was used to systematically evaluate all pairwise interactions. The effect of MRSA infections on hospital stay duration was assessed with Cox proportional hazards models with adjustment for independent risk factors of MRSA. The impact of MRSA infections on fatality was evaluated by logistic regression analysis (MRSA versus MSSA) and exact logistic regression analysis (MRSA versus non-*S*. *aureus*), with adjustment for variables that had *P* values < 0.1 in the univariate MRSA predictor analysis. A two-tailed *P* value below 0.05 was considered statistically significant. All statistical analyses were performed using Stata software, version 13.0 (College Station, Texas).

## Results

### Patients

A total of 57 patients with MRSA isolates, 116 patients with MSSA isolates and 102 patients without *S*. *aureus* isolates were included in this study. The most common *S*. *aureus* specimen was sputum (97/173, 56.1%), followed by abscess (45/173, 26.0%). The neurology department ranked first in proportion among the wards that the patients hospitalized in (41/173, 23.7%), followed by the dermatology department (35/173, 20.2%).

### Antimicrobial susceptibility testing

Of the *S*. *aureus* strains evaluated, 92% (160/173) displayed resistance to penicillin, 90% (156/173) to ampicillin, 78% (134/173) to levofloxacin, 54% (94/173) to erythromycin, 45% (78/173) to tetracycline, 39% (67/173) to clindamycin, 35% (61/173) to ciprofloxacin, 33% (57/173) to oxacillin, 27% (47/173) to amoxicillin, imipenem and cefazolin, 27% (46/173) to gentamicin, 13% (23/173) to chloramphenicol, 9% (15/173) to sulfamethoxazole/trimethoprim, 3% (6/173) to rifampicin, 2% (4/173) to quinupristin/dalfopristinand, and 0% (0/173) to linezolid and vancomycin. According to chi-square test, the resistance rates for individual agents were significantly higher in the MRSA group than in the MSSA group, with the exception of linezolid, vancomycin, levofloxacin and quinupristin/dalfopristin (Table 1).

**Table 1:** Comparison of antimicrobial susceptibility between MRSA and MSSA.

Drug agent	MRSA (n = 57)	MSSA (n = 116)	*P*
	No. (%) resistant isolates	No. (%) resistant isolates	
GEN	38 (66.67)	8 (6.90)	<0.001
IMI	47 (82.46)	0 (0.00)	<0.001
CIP	44 (77.19)	17 (14.66)	<0.001
LEV	49 (85.96)	85 (73.28)	0.060
PEN	57 (100.00)	103 (88.79)	0.001
VAN	0 (0.00)	0 (0.00)	NA*
LIN	0 (0.00)	0 (0.00)	NA*
AMO	47 (82.46)	0 (0.00)	<0.001
AMP	56 (98.25)	100 (86.21)	0.012
OXA	57 (100.00)	0 (0.00)	<0.001
S/T	15 (26.32)	0 (0.00)	<0.001
CLI	42 (73.68)	25 (21.55)	<0.001
Q/D	3 (5.26)	1 (0.86)	0.081
RIF	5 (8.77)	1 (0.86)	0.009
CHL	14 (24.56)	9 (7.76)	0.002
TET	45 (78.95)	33 (28.45)	<0.001
CEF	47 (82.46)	0 (0.00)	<0.001
ERY	53 (92.98)	41 (35.34)	<0.001

* NA: Not Available.

GEN: gentamicin; IMI: imipenem; CIP: ciprofloxacin; LEV: levofloxacin; PEN: penicillin; VAN: vancomycin; LIN: linezolid; AMO: amoxicillin; AMP: ampicillin; OXA: oxacillin; S/T: sulfamethoxazole/trimethoprim; CLI: clindamycin; Q/D: quinupristin/dalfopristin; RIF: rifampicin; CHL: chloramphenicol; TET: tetracycline; CEF: cefazolin; ERY: erythromycin.

### Risk factors

Gender, age, underlying conditions, invasive procedures, ICU stay, surgery, and history of hospitalization and antibiotics usage were included as potential influencing factors for MRSA acquisition. The results of the multivariable logistic analyses of risk factors for MRSA infections are shown in Table 2. Three or more prior hospitalizations, chronic obstructive pulmonary disease, and use of a respirator were independently and statistically associated with MRSA infections in comparison to MSSA infections (pseudo R^2^ = 0.16, area under ROC curve = 0.72). Use of a respirator and tracheal intubation were identified as risk factors for MRSA infections in comparison to *S*. *aureus* negative patients (pseudo R^2^ = 0.09, area under ROC curve = 0.62). No statistically significant interactions between any risk factors were found.

**Table 2:** Multivariate logistic regression analysis of risk factors for MRSA infections.

Risk factor	MRSA versus MSSA		MRSA versus *S*. *aureus* (-)	
	aOR (95% CI)	*P*	aOR (95% CI)	*P*
Prior hospitalizations≥3	2.8 (1.3–5.8)	0.007		
COPD	5.9 (1.7–20.7)	0.006		
Respirator	3.6 (1.0–12.9)	0.046	3.8 (1.0–13.9)	0.047
Tracheal intubation			8.2 (1.5–45.1)	0.016

aOR: adjusted Odds Ratio; COPD: Chronic Obstructive Pulmonary Disease.

### Clinical outcomes

**Fig 1:**
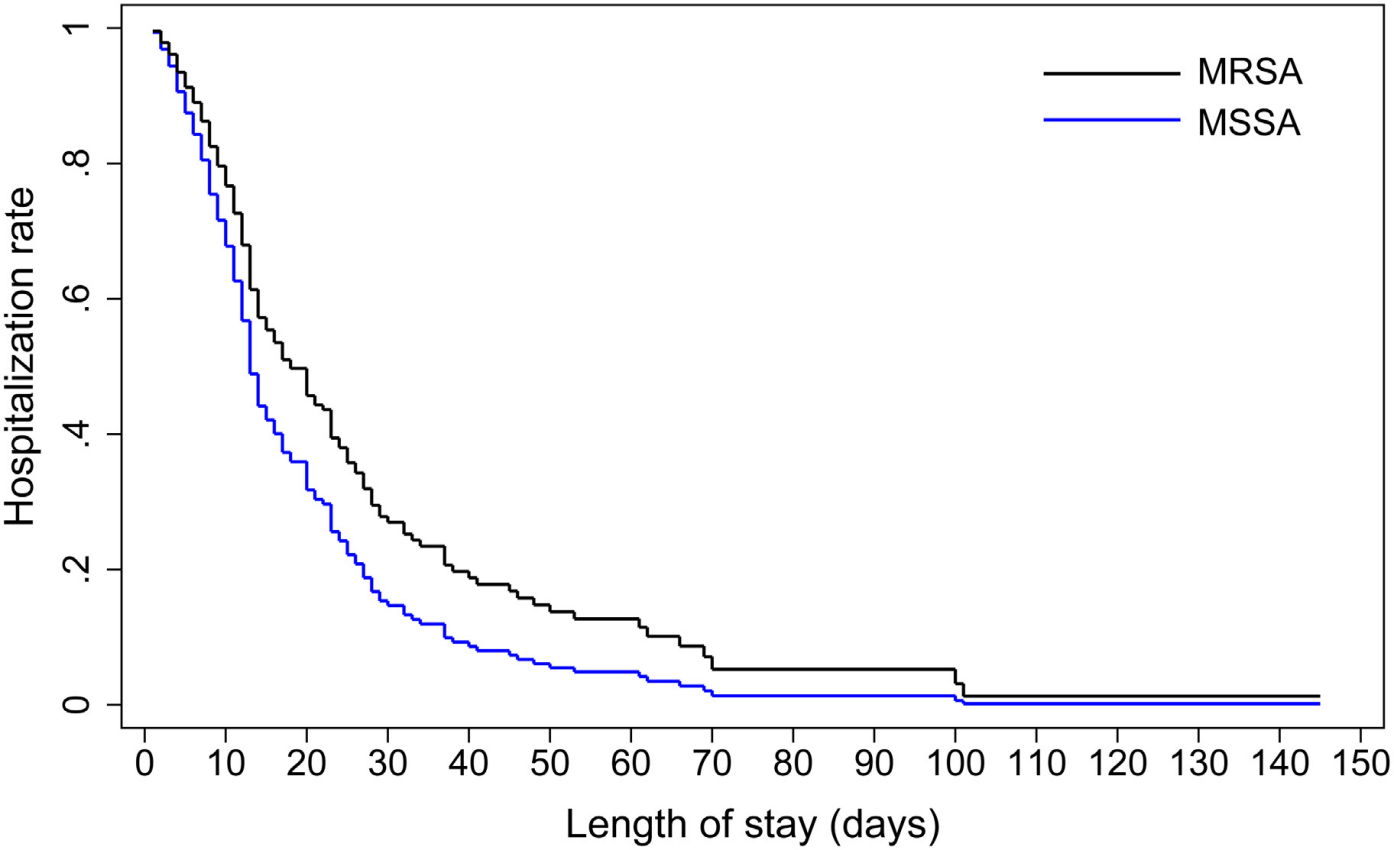
Comparison of hospitalization rates for patients with MRSA infections and those with MSSA infections. Adjusted for three or more prior hospitalizations, COPD and respirator use.

**Fig 2:**
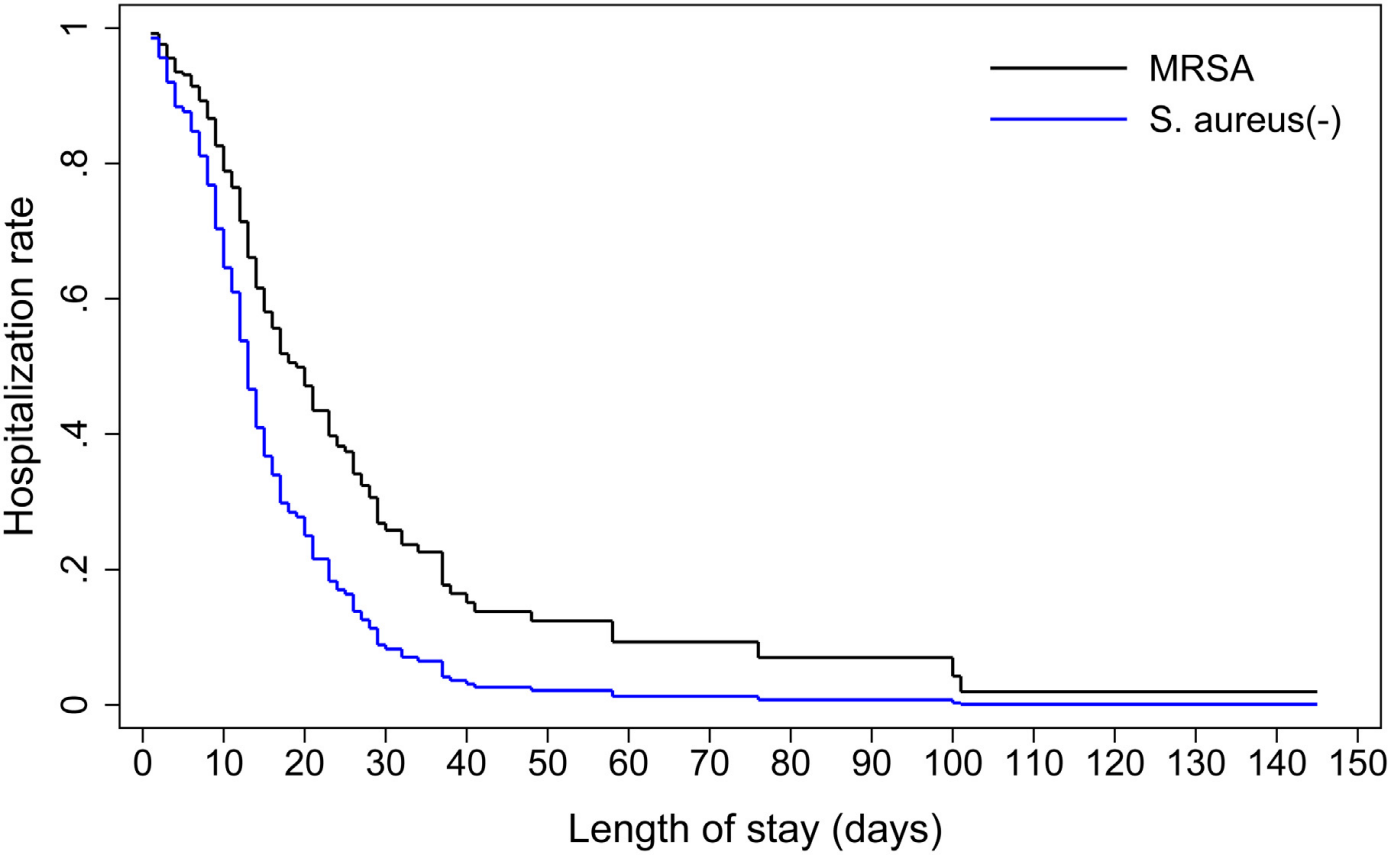
Comparison of hospitalization rates for patients with MRSA infections and those without *S*. *aureus* infections. Adjusted for respirator use and tracheal intubation.

Cox proportional hazards regression models were introduced to examine the possible relationships between MRSA infections and length of hospital stay. The patients who died during hospitalization were censored in the model. MRSA infections were associated with a longer duration of hospitalization, and this association was still significant after adjusting for other covariates (MRSA versus MSSA: Table 3 and Fig 1; MRSA versus non-*S*. *aureus*: Table 4 and Fig 2). The fatality rates of the MRSA, MSSA and *S*. *aureus* negative groups were 21% (12/57), 8% (9/116) and 0% (0/112), respectively. MRSA infections were associated with increased risk of fatality (MRSA versus MSSA: adjusted OR 2.7, 95% CI 1.0–7.0, pseudo R^2^ = 0.08, area under ROC curve = 0.71; MRSA versus *S*. *aureus* negative, adjusted OR 31.6, 95% CI 4.4-inf, model score = 26.41).

**Table 3:** Cox proportional hazards analysis of the association between MRSA infections and length of hospital stay (compared with MSSA).

Variable	Univariate Model		Multivariate Model	
	HR (95% CI)	*P*	HR (95% CI)	*P*
MRSA	1.8 (1.2–2.5)	0.002	1.5 (1.0–2.2)	0.046
Prior hospitalizations≥3	1.5 (1.1–2.1)	0.025	1.3 (0.9–1.9)	0.156
COPD	1.2 (0.7–2.1)	0.468	0.9 (0.5–1.7)	0.840
Respirator	2.2 (1.2–3.9)	0.010	1.7 (0.9–3.2)	0.090

HR: Hazard Ratio; COPD: Chronic Obstructive Pulmonary Disease.

**Table 4:** Cox proportional hazards analysis of the association between MRSA infections and length of hospital stay (compared with non-*S*. *aureus*).

Variable	Univariate Model		Multivariate Model	
	HR (95% CI)	*P*	HR (95% CI)	*P*
MRSA	2.2 (1.5–3.2)	<0.001	1.8 (1.2–2.7)	0.002
Respirator	2.2 (1.2–3.9)	0.007	1.4 (0.7–2.6)	0.310
Tracheal intubation	2.7 (1.3–5.5)	0.007	1.8 (0.8–3.8)	0.143

HR: Hazard Ratio.

## Discussion

*S*. *aureus* is among one of the six most infamous ESKAPE pathogens that easily develop antibiotic resistance (*Enterococcus faecium*, *Staphylococcus aureus*, *Klebsiella pneumoniae*, *Acinetobacter species*, *Pseudomonas aeruginosa and Enterobacter species*) [13]. MRSA infections were recognized as a major threat to healthcare settings in China [14, 15]. To the best of our knowledge, this study is the first to provide comprehensive data on the antimicrobial resistance of clinical MRSA isolates, the risk factors for MRSA infections, and the hospital stay duration and fatality of MRSA infected individuals in a tertiary care hospital in Guangzhou, China.

*S*. *aureus* isolates displayed remarkably high rates of resistance to penicillin and ampicillin relative to the other antibiotics tested, which is in accordance with previous reports [16, 17]. The possible complex molecular mechanism of resistance to these drugs involves circumvention of the mechanism of action of penicillin, which is its binding protein 2a (PBP2a), a gene product of the *mec*A gene that is involved in cell wall biosynthesis [18]. A recent study also demonstrated that some isolates have evolved from MSSA to MRSA due to antibiotic exposure, which highlights the significance of appropriate antibiotics usage in clinical settings [19]. We did not find any strains resistant to vancomycin and linezolid, indicating those antibiotics are still effective for treating *S*. *aureus* healthcare-associated infections. The resistance rates of most agents were higher in the MRSA group than in the MSSA group, so it is necessary to implement strategies to reduce the ratio of MRSA infections to MSSA infections in healthcare settings.

The multivariate analysis indicated that chronic obstructive pulmonary disease is an independent risk factor for MRSA infections. An association between underlying illness and MRSA infections has also been suggested by recent studies [20–22]. Use of a respirator and tracheal intubation were also confirmed as independent risk factors for MRSA acquisition. This was not surprising, given that previous reports have obtained similar results regarding the role of invasive procedures in the drug resistance of *S*. *aureus* [23, 24]. In addition, we found that a history of multiple hospitalizations was statistically associated with MRSA infections, which is similar to the findings of another recent report [25]. Therefore, more intensive infection control measures should be implemented for patients with these risk factors.

We quantitatively evaluated the impact of MRSA infections on clinical outcomes and found that it resulted in a longer hospital stay and higher fatality in comparison to the two control groups. The results agree with those of some previous meta-analyses conducted in various countries and regions [6, 26, 27]; those studies detected a positive relationship between MRSA infections and poor prognosis.

Our research has certain strengths. First, we employed a case-control-control study design, and this two parallel case-control design greatly reduces the selection bias caused by the use of a single control group and improves the accuracy of our results. Furthermore, we selected all cases and controls from one tertiary care hospital so that differences between institutions, which are potential confounders, were avoided. Moreover, our analyses were mainly based on the electronic medical records review instead of self-report questionnaires, which reduces recall bias to ensure the accuracy of our data.

As with any retrospective study, our study also bears some limitations. First, we could not follow patients after discharge, because our data sources were electronic medical records, so we could not rule out the possibility that some discharged patients may have had a reoccurrence of the healthcare-associated infections or even died as a result of them. Furthermore, some typos may have been present in the electronic medical records, and it is difficult to observe and correct those errors through retrospective inspection of the records. In addition, the sample size was small, which may have reduced the statistical power of our results and therefore negatively impacted the value of our findings.

In summary, MRSA had greater resistance rates for the majority of antibiotics tested when compared to MSSA. Three or more prior hospitalizations, chronic obstructive pulmonary disease, use of a respirator and tracheal intubation were identified as independent risk factors for MRSA infections. The acquisition of MRSA infections resulted in a longer hospital stay and increased fatality.
